# Observations on Infantile Remittent Fever

**Published:** 1818-05

**Authors:** Robert Smith

**Affiliations:** Resident Physician at Maidstone, in Kent


### *Observations on Infantile Remittent Fever.*

By Robert Smith, M. D.*

Resident Physician at Maidstone, in Kent.

WHEN we reflect that no less than one half of the human race is cut off under ten years of age, it must be apparent that the diseases of childhood, affecting as they do, at once, the happiness of domestic privacy and the prosperity of the commonwealth, form a most interesting subject of inquiry to the political as well as the medical philosopher. It is to be lamented, however, that the attention of the latter has not been turned, till of late years, towards this branch of practice. An erroneous idea long obtained among some of the most distinguished of the Faculty, that the diseases of infancy, when not in themselves slight, are unattended by those specific symptoms, or marked circumstances, which can guide us in establishing a correct diagnosis, or a rational mode of cure. Hence an insufficient attention was given to them, and the remedies employed were generally inert, and sometimes even hurtful.

As a natural consequence of this supineness of professional men, the management of children in sickness generally fell to old women, nurses, or empirics—persons altogether unqualified for so delicate a task. It would be endless to recount the various fatal blunders which have been committed by their confounding one disease with another, by their timidly and ignorantly allowing a dangerous disease to go on until relief came too late, by their exhibiting improper or dangerous remedies. On the latter head we ought ever to remember, that such unskilful persons do not always confine themselves to rhubarb, magnesia, and other simples; but too often, in the rashness of ignorance, administer spirituous liquors and opiates, in their various forms; substances which, by repeated experience, are ascertained to be fraught with the utmost danger to the infantile constitution.†

The diseases of children, for the most part, have greater simplicity of cause than those of adults, because, in the

* Translated and condensed from his valuable Inaugural Dissertation, published at Edinburgh in August last.

† In this sentence of reprobation, Dalby's Carminative (which, we believe, is a preparation of assafœtida) ought also to be included; for Dr. Clarke, at p. 33 of his excellent work, says, he has seen 40 drops of it prove fatal, in a few hours, to very young infants. Edit.

former, the phænomena of disease are generally naked, as it were, and are not exasperated, modified, or altered, by the influence of those mental causes, which produce such various impressions on the diseases of the latter.

The symptoms also are few and simple; and though the little patient cannot himself describe them, still his geneneral appearance, the tone of his voice, the state of the respiration and of the pulse, the cast of countenance, the state of the pupils, the various gestures, and the condition of the different secretions and excretions, will, to the practical physician, afford a juster and more accurate knowledge of the malady, than even the most elaborate description of his older and wiser patients; descriptions which are too often contaminated or warped by their own silly notions of pathology, or mixed up with the natural exaggeration of their hopes or fears.

As it may be of some consequence to ascertain the identity of the disease I am treating of, I may be permitted to say, that it is the "worm-fever" of Musgrave; the "febris synochus puerorum" of Mercurialis; the "febris ardens continua infantum" of Timæus; the "fievre ardente des enfans" of Lieutaud; the "hectica infantilis" of Sauvages; the "hectica febris infantum" of Sydenham; and "febris lenta infantum" of Hoffman.

All these authors, together with Armstrong and others, have employed their pens on this malady; but it is to Dr. Butter, of Derby, that we are indebted for the first accurate and elegant treatise on the subject: it was published in 1782. The following is the definition given by that celebrated physician:

"Exacerbationes somniculosæ, remissiones vigiles, capitis alvique dolor, anorexia, sitis haud magna, et dejectiones mucosæ-fœtidæ, febrem infantilem remittentem designant."

Infantile remittent fever attacks children between their first year and their tenth or twelfth. This attack is seldom sudden; on the contrary, it is generally very insidious and obscure; so much so, indeed, that often the first circumstances that attract the notice of the parents or nurses are, slow fever in the evenings, nausea, restlessness, and sometimes even delirium.

It is never contagious, as has been well observed by Dr. Heberden;* at least I have seen no instance countenancing a suspicion of that nature.

---

* Heberd. Morbor. Puerilium Epitome, p. 56.

The little patient at first appears uneasy, restless, and morose, without any evident cause. He loses all relish for his usual diversions and his usual play-fellows, and desires only to repose in his mother's lap, or lie down in bed. Short dry cough, accelerated respiration, anorexia, vomiting of the food unchanged which had recently been taken, flushed face, heated and dry skin, swollen and tense abdomen, are generally observed at the onset. The bowels are generally confined, but sometimes loose, and the alvine evacuations are fœtid, and of a morbid colour. The tongue is whitish or brownish. Urine variable, being one while small in quantity and thick, and at other times high coloured and copious: now and then, according to Dr. Butter, " it puts on a milky appearance almost as soon as made."

By and bye, all these symptoms remit; but, although the child returns to its accustomed sports, the pulse continues quicker and the skin drier than usual. On the following day, the paroxysm returns, ushered in by coldness of the extremities; and exacerbations more and more severe supervene. At last they become so aggravated in severity and duration, that towards the close of the disease the remissions are hardly perceptible.

Under these circumstances, all the symptoms above enumerated become worse. The pulse is 150 or 170, and often rather hard. The little sufferer picks his nose, lips, ears, and eyes; rubs his face and head; is soporose, grinds his teeth, and moans during sleep, from which he often awakes suddenly as if frightened. The belly becomes more painful, and is obstinately costive; or else the diarrhœa, which had previously been present, is much exasperated. The stools are blackish or glutinous like bird-lime, and sometimes resemble curdled milk floating in whey, while at other times they are green and mucous, or chalky, and always most offensive in smell. Lumbrici are also occasionally found in the intestines.

Convulsions attack different parts of the body; and not unfrequently partial paralysis takes place. Constant delirium or coma generally precedes death; but, in some instances, the child, extenuated by the long duration of the disease, sinks from no other apparent cause than debility.

When a favourable crisis takes place, the exacerbations become milder and shorter, the pulse slower, the belly more natural, and the sleep more calm and refreshing.

Dissection has hitherto thrown no light on the pathology of this disease. One case was examined after death by Dr. Pemberton, and another by Dr. Armstrong; but the only thing these gentlemen found worthy of notice was, considerable fæcal infarctions in the intestines, particularly in the colon.

The causes of this fever are, whatever either directly or indirectly debilitates or *irritates* the system in general or the chylo-poietic organs in particular. When once fairly established, it is attended with very considerable danger, contrary to the opinion of Dr. Underwood, who, at p. 152 of his work, pronounces it comparatively a harmless complaint. When pus is passed by stool, it is a very bad symptom, as indicating a morbid state of the mesenteric glands.

It is a matter of great importance, but of the utmost difficulty, to distinguish infantile remittent fever from difficult dentition, but more especially from acute hydrocephalus. In the former, the state of the mouth and gums will materially assist us in forming a judgment (though it should ever be borne in mind that painful dentition either attends upon, or often ends in, infantile remittent fever); but in the latter (acute hydrocephalus to wit), the line of demarcation is so narrow, and generally so obscure, that it requires long experience and accurate observation to make a just diagnosis. A very slight inspection of the records of modern surgery will show how often men of the first-rate talents have been led into error on this point. In fact, the fever I speak of, sometimes terminates in hydrocephalus; besides, it is well known that there are many symptoms common to both diseases, such as the insidiousness of the attack, the state of the respiration, of the temperature, and of the stomach and bowels, the convulsions, delirium, coma, &c. In short, there is not a single symptom occurring in hydrocephalus, but what will also be found to belong to infantile remittent fever, even strabismus and dilated pupils, which are held to be more especially characteristic of the former, sometimes (though rarely, it is true), appear in the latter disease. In confirmation of these remarks about the strong resemblance of the two complaints, and the almost utter impracticability of an accurate diagnosis, the reader has only to consult the works of Clark, Burns, Cheyne, Armstrong, Rush, Underwood, and Heberden.

The last-mentioned venerable and most admirable physician, after ably describing the customary symptoms of acute hydrocephalus, uses the following words: "Neque tamen perpetuum est ut hæc signa ex hydrope cerebri nascantur, etenim vidi ea omnia in puero cujus cerebrum non nisi consueta humoris copia humectatum erat, teste peretissimo anatomico, qui cadaver inciderat." (Comment. de Morb. Hist. et Cur. p. 186).

Yet, in my own opinion, difficult as the diagnosis certainly is, a wrinkling and knitting of the eye-brows, an aversion to light, contraction of the pupils, an increased acuteness of hearing and touch, and a degree of heat in the head greater than that of the rest of the body, may, in the first days, be reckoned as the surest indications of hydrocephalus, in contra-distinction to infantile fever. Again, dilatation of the pupils, strabismus, slow and intermitting pulse, obstinate costiveness, screaming, &c. sufficiently point out the advanced and hopeless stage of the disease.

Dr. Pemberton, in his excellent practical Treatise on the various Diseases of the Abdominal Viscera, p. 166, speaks in the following terms: "In the delirium of hydrocephalus internus, the faculties are totally destroyed, and the muttering ravings of the patient are without sense or reason, and from this state he cannot be roused, so as to command his attention to any object, even for the shortest period. But in the other species of delirium (infantile remittent fever), the child, during this state, can at any time be recalled to his senses, which he will retain for a few minutes, acting and talking consistently."

The means of cure in the remittent fever of infants may be comprised under the four following indications:—1. To remove the exciting causes. 2. To diminish febrile excitement, and to relieve local or adventitious symptoms. 3. To restore the action and secretions of the primæ viæ to their healthy state; and, 4. To restore the tone of the constitution.

With regard to the first indication, it will be highly proper, as a preliminary measure, in every instance where teething is not completed, to practice very free scarification of the gums at those points where any degree of redness or fulness is apparent. Then an emetic of ipecacuanha wine or powder, or of wine of tart. antimonii*

* Dr. Smith seems to consider it a matter of indifference whether the one or the other of these substances be employed as an emetic. But we beg to state, that Dr. Hamilton, Professor of Midwifery in the University of Edinburgh, whose talents and unlimited experience in the diseases of women and children are well known, strongly reprobates the use of *antimonial* emetics in infantile diseases, from their frequently producing alarming symptoms, and even death.—We would also refer our readers to what M. Orfila has said on this subject, in his book on Toxicology. ED.

is to be given in divided doses till it operates. When the vomiting is over, and the stomach has again become settled, the bowels are to be briskly opened by a full dose of scammony, jalap, or (when the irritable state of the stomach will not bear these) calomel, followed up in three or four hours by small doses of neutral salts, infusion of senna, or the like.

When the stomach is very irritable, a glyster should be administered; indeed, in any case, this should never be omitted, as it removes indurated fæces, and, as an internal fomentation, mitigates pain and irritation.

The second indication is to be fulfilled by the use of the tepid bath (or even the cold bath, if the heat is urgent) twice a-day. The water should be acidulated with vinegar. The bath has most marked effects in subduing inordinate irritation and heat, and in producing sleep. When the abdomen is tense and very painful, it should be fomented with decoction of camomile and white poppy heads, and rubbed with camphorated or anodyne liniments. Emollient enemata and flannel rollers are also highly useful: in some cases, even the internal use of a few drops of tinct. opii is called for.

Where the head is much affected—where its temperature is greater than that of the rest of the body, and other marks of sanguineous determination to the brain are present, the case should be treated as one of real hydrocephalus, and blood should freely be drawn by leeches, by cupping, or by opening either a branch of the temporal artery, or the external jugular vein. In every doubtful case, this, to say the least of it, is at any rate *safe* practice.

When loss of speech, or other paralytic affections take place, they can only, in my opinion, be removed by general treatment. I have often witnessed symptoms of this kind gradually go off as the healthy action of the stomach and intestines returned.

3d. For restoring the healthy actions and secretions of the chylo-poietic viscera, nothing seems so efficacious as small, and oft repeated doses of calomel, followed up, after some hours, by a cathartic of salts, senna, or oleum ricini. Sometimes, when the bowels are very irritable, small quantities of mist. cretac. cum tinct. opii must be given alternately with the purgatives above specified.

Even when the disease is almost completely got under, submuriate of mercury, with the occasional interposition of a dose of neutral salts, should be given every night, or morning and evening, as may seem most fit, until the natural secretions, and the natural action, of the chylopoietic viscera be completely re-established.

It is almost unnecessary to say, that the little patient should sleep with his head well raised, in a chamber where cool air is freely admitted. Indeed, as to the latter point, not only the admission of cool air, but every part of the antiphlogistic regimen should be enforced during the whole progress of the disease. During convalescence, the diet should be increased only by very slow degrees.

The fourth indication, namely, to restore the tone of the constitution, is best accomplished by change of air, and a diet accommodated to the existing state of the digestive organs. With these means, may advantageously be conjoined moderate exercise, bitter infusions, or preparations of iron.

It appears to me, that wine* is a remedy by no means suited to the diseases of children: nay, I have seen it positively hurtful. If it is to be used, it should be with the utmost caution, and largely diluted with water.

---

* In this last remark of Dr. Smith, we most heartily concur, because we know his experience in the diseases of children has been very ample. We may take this opportunity of reprobating the pernicious custom, practised by some parents who, in every other respect, are most intelligent, of giving every day a glass of Port wine to a child of two or three years old. To every one who knows any thing about the human œconomy, it must be self evident, that the tender digestive organs of a child cannot assimilate such strong potations; and we think the veto against giving "strong drink to babes," is no less judicious in a physical, than it is in a moral sense.

But the ruinous effects of wine on the digestive organs are not all; the ulterior traces of the poison develope themselves on the tender structure of the infant brain, by causing sanguineous impulse in that important part. Putting the danger of hydrocephalus, &c. on one side, we really do not know a likelier method of making children either epileptics or dunces, than a pernicious and unnatural custom of this kind.

We have often regretted to see that professional men, though they may not expressly sanction the practice, give a sort of reluctant consent to it, from the importunities of the mother about the necessity of "making her infant strong," instead of firmly and decisively setting their face against this off-shot of modern luxury, which threatens, like a noxious weed, to choak the growth of health, virtue, talents, and all that is "lovely or of good report," in the child's future progress through life. EDIT.

